# Medical Examiner and Western Medical Schools

**Published:** 1844-08

**Authors:** 


### MEDICAL EXAMINER AND WESTERN MEDICAL SCHOOLS.

We must say that we consider the allusion to *Western Medical Schools* by the editor of the Medical Examiner, in the number of that paper for June 29th, an unfortunate one. We hope without intending it, he has made a charge againsthis brethren in this region which must give general and deep offence. A distinguished writer and teacher on this side of the mountains has felt himself called upon to enter his protest against the charge of the Medical Examiner, and to defend western schools of medicine against the imputation of having deteriorated the profession in the Valley of the Mississippi. In the performance of this task he has indulged in a severity of remark which we should avoid; but it will not be denied by any one who has read his defence, that it is one of singular ability. We make room for a few extracts from it. Speaking of Western Medical Schools, the writer says:

"Nor do we hesitate to assert, that, in them the teaching is as well planned and extensive, and as thoroughly and efficiently executed, as any other schools in the United States. In proof of all this, the pupils of Western schools, when under examination, acquit themselves as creditably as do those of Eastern schools, in every branch of medicine, and, on the treatment of western fevers, much more so.

"The gentleman, as we have already seen, utters, midway between sighs and tears, a piteous jeremiad, because, in his own words, 'since the establishment of medical schools in the Valley of the Mississippi, comparatively few young men from that quarter seek instruction *elsewhere*,' For 'elsewhere,' read *in* the JEFFRSON MEDICAL COLLEGE, and the meaning of the clause will be correctly told. For the only source of his lamentation is, not that medicine is less efficiently taught than formerly; but that the pupils of the West and South have, in a great measure, ceased to seek their education in the medical schools of the East. Why? Because they receive in the institutions of their own region, on easier terms, an education in *all respects* as good, and in some much better than they can possibly receive in the Atlantic schools.

"It is true, that a certain 'teacher in the south-west,' whom the Philadelphia Professor might as well have named, has long and earnestly inculcated the doctrine, that difference in climate, locality, soil, pursuit, habit, and diet, is productive of a corresponding difference in the diseases of man, as well as in those of the inferior animals, which imperatively demand a different mode of treatment; and that that mode can be learnt only by *observation* and *experience*. But it is equally true, that the doctrine did not originate with that teacher. Far otherwise.

"To allege that, previously to any experience in it, a physician can treat successfully a bold and malignant form of fever, bespeaks a degree of *ignorance* or *weakness*, or of an amalgamation of the two of which none but a medical ignoramus or intentional deceiver, can be suspected. As well may it be contended, that a young graduate in medicine, who has obtained his degree, by reading and attending lectures alone, without having ever visited a sick-room, or the ward of a hospital, is fully ripe for clinical practice. Yet is it a familiar fact that such a youth is more incompetent to the treatment of a severe and dangerous case of fever, than a moderately well-trained nurse. Even Sydenham has recorded the fact, that, on the explosion of a new and severe epidemic, in London, of whose diseases he had the knowledge of a life-time, he was too often unsuccessful in his treatment of the *first few cases* of it. Of that great luminary in medicine, therefore, *observation* and *experience* were *necessary instructors*. And. in relation to himself, Dr. Rush did not hesitate to make a like acknowledgment. When yellow fever moreover made its appearance in Philadelphia, in 1793, every honest and respectable practitioner in the place, was fully prepared to make the same avowal." Y.

